# Intersection between Redox Homeostasis and Autophagy: Valuable Insights into Neurodegeneration

**DOI:** 10.3390/antiox10050694

**Published:** 2021-04-28

**Authors:** Hyungsun Park, Jongyoon Kim, Chihoon Shin, Seongju Lee

**Affiliations:** Program in Biomedical Science & Engineering, Department of Anatomy, College of Medicine, Inha University, Incheon 22212, Korea; hyungsun@inha.edu (H.P.); jyoon5004@inha.edu (J.K.); promise0514@inha.edu (C.S.)

**Keywords:** autophagy, redox homeostasis, ROS, neurodegenerative diseases

## Abstract

Autophagy, a main degradation pathway for maintaining cellular homeostasis, and redox homeostasis have recently been considered to play protective roles in neurodegenerative diseases such as Alzheimer’s disease, Parkinson’s disease, and amyotrophic lateral sclerosis. Increased levels of reactive oxygen species (ROS) in neurons can induce mitochondrial damage and protein aggregation, thereby resulting in neurodegeneration. Oxidative stress is one of the major activation signals for the induction of autophagy. Upon activation, autophagy can remove ROS, damaged mitochondria, and aggregated proteins from the cells. Thus, autophagy can be an effective strategy to maintain redox homeostasis in the brain. However, the interaction between redox homeostasis and autophagy is not clearly elucidated. In this review, we discuss recent studies on the relationship between redox homeostasis and autophagy associated with neurodegenerative diseases and propose that autophagy induction through pharmacological intervention or genetic activation might be a promising strategy to treat these disorders.

## 1. Introduction

Neurodegenerative diseases are a heterogeneous group of disorders characterized by progressive loss of the structure or function of neurons. The major risk factor for the development of neurodegenerative diseases is aging; therefore, the prevalence of these disorders increases as the average lifespan increases. However, effective treatments for most neurodegenerative diseases are still lacking. Over the past decades, various studies demonstrated that the progression of neurodegenerative diseases is accompanied by decreased antioxidant levels, increased oxidative stress, and decreased autophagy. Oxidative stress can induce autophagy, which relieves the cytotoxicity caused by accumulated oxidative stress. Here, we describe the dysregulation of redox homeostasis that is observed in major neurodegenerative diseases, including Alzheimer’s disease, Parkinson’s disease, and amyotrophic lateral sclerosis. We then discuss the molecular pathways related to oxidative stress and autophagy in neurodegenerative diseases. Finally, we suggest that manipulation of autophagy might be an effective strategy to alleviate oxidative stress in patients suffering from neurodegenerative diseases.

## 2. Main Players Involved in Maintaining Redox Homeostasis

Cells continually generate small, highly reactive molecules called reactive oxygen species (ROS) due to incomplete reduction of oxygen during aerobic metabolism or immune response. ROS include superoxide anion (O_2_^●−^), hydrogen peroxide (H_2_O_2_), hydroxyl radical (^●^OH), and 4-hydroxynonenal (HNE). ROS can be generated from endogenous sources. The mitochondrial electron transport chain is recognized as one of the major modes of cellular ROS generation [1]. NADPH oxidases, which share the capacity to transport electrons across the plasma membrane, are also able to generate superoxide and other downstream ROS [2]. Cytochrome P450 generates ROS and lipid peroxidation products which might interact with DNA to create oxidative DNA adducts [3]. ROS generally cause detrimental effects, such as DNA damage, lipid peroxidation, and protein oxidation, in cells, ultimately leading to cellular stress or pathological conditions. Therefore, under normal physiological conditions, the formation and elimination of ROS are tightly regulated by endogenous antioxidants and ROS scavengers, to maintain homeostasis and avoid oxidative stress and toxicity. The antioxidants that are produced endogenously, include superoxide dismutase (SOD), catalase (CAT), glutathione (GSH), and peroxiredoxins (Prxs). When electrophiles, such as ROS, are increased in turn, antioxidant responses, such as NF-κB or Keap1-Nrf2-mediated signaling cascade, are upregulated to restore the original steady-state [4]. These redox reactions are important for cellular homeostasis to regulate cell signaling, proliferation, and survival [5].

Superoxide anion (O_2_^●−^) is produced by one-electron reduction of dioxygen, which occurs in the mitochondrial electron chain reaction or NADPH oxidase-mediated immune response in phagocytic cells [6]. Owing to its high reactivity, the generation of O_2_^●−^, even at low levels, is essential for the oxygen-dependent killing of invading pathogens in phagocytic cells [7]. However, there is a proper defense mechanism to remove O_2_^●−^, as it is harmful to cells when present in excess. SOD is an antioxidant enzyme that catalyzes the dismutation of O_2_^●−^ into molecular oxygen and hydrogen peroxide, which is the first defense mechanism against ROS. Almost all organisms that use O_2_^●−^ for mitochondrial respiration express SOD. In mammals, SODs exist in three isoforms with distinct compartmentalization—SOD1 in the mitochondrial intermembrane space and cytoplasm, SOD2 in the mitochondrial matrix, and SOD3 in the extracellular region [8].

Hydrogen peroxide (H_2_O_2_) has an unstable peroxide bond, which can oxidize proteins, lipids, and DNA. Mitochondrial respiratory chain is a continuous source of H_2_O_2_ resulting from the dismutation of O_2_^●−^ by SOD [9]. Upon H_2_O_2_ generation, CAT rapidly detoxifies it to molecular oxygen and H_2_O [10]. CAT, which is enriched in peroxisomes, contains four iron-containing heme groups that allow it to react with H_2_O_2_. Glutathione system also eliminates H_2_O_2_ from organisms [11]. H_2_O_2_ is reduced by glutathione peroxidase, which transfers the energy of the peroxide to GSH, a small sulfur-containing tripeptide. Since glutathione exists in reduced (GSH) and oxidized (GSSG) states, the ratio of GSH to GSSG can be a sensor of oxidative stress [12]. As H_2_O_2_ has a lower activity than the hydroxyl radical, it remains in the nucleus for a longer time, which further damages DNA. Although H_2_O_2_ has cytotoxic effects, it also acts as a signaling molecule in regulating various biological processes, such as immune cell activation and vascular remodeling [13]. Therefore, its production is highly regulated as part of redox homeostasis.

Hydroxyl radical (^●^OH), the three-electron reduction state of O_2_^●−^, is an extremely reactive and short-lived ROS. It is produced by reduction of H_2_O_2_ or due to the immune responses in macrophages and microglia [14]. The hydroxyl radical damages almost all types of molecules, such as nucleic acids, lipids, and amino acids. In addition, the hydroxyl radical attacks the plasma membrane, causing membrane damage. Due to its high reactivity, the hydroxyl radical cannot be used as a substrate for any enzyme and therefore cannot be removed by enzymatic reactions [15]. The hydroxyl radical is neutralized by toxic reactions with adjacent oxidizable molecules.

Lipid peroxidation is a process that occurs when free radicals attack nucleophilic polyunsaturated fatty acids in lipids. Since lipids are particularly abundant in the cell membrane, lipid peroxidation leads to membrane lipid degradation and cell damage. It produces lipid hydroperoxides (LOOH), such as malondialdehyde, 4-hydroxynonenal (HNE), 4-oxo-2-nonenal, and acrolein, thereby causing oxidative stress. For example, modification of mRNA expression or the activity of antioxidant enzymes increases, as the amount of HNE increases due to lipid peroxidation [16]. Thus, HNE is often regarded as a marker of oxidative stress.

**Figure 1:**
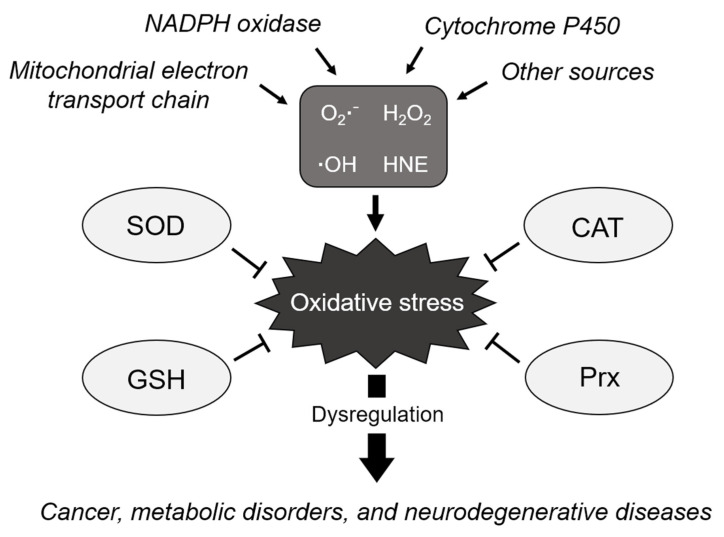
Schematic of the biological processes regulating cellular redox homeostasis. ROS are generated from various sources due to incomplete reduction of oxygen, causing oxidative stress. The elimination of ROS is tightly regulated by antioxidants, such as SOD, to maintain homeostasis and avoid oxidative stress. However, dysregulation of oxidative stress induces various diseases such as cancer, metabolic disorders, and neurodegenerative diseases. O_2_^●−^: superoxide anion, H_2_O_2_: hydrogen peroxide, ^●^OH: hydroxyl radical, HNE: 4-hydroxynonenal, SOD: superoxide dismutase, CAT: catalase, GSH: glutathione, and Prx: peroxiredoxin.

The accumulation of ROS, resulting from the failure to maintain redox homeostasis, permanently induces increased oxidative stress, thereby leading to the functional loss of proteins, cellular apoptosis, mutagenesis, carcinogenesis, and fibrosis [17,18,19]. Increased ROS levels are associated with oxidative DNA damage and genomic instability [20]. NADPH oxidase-derived ROS were identified as the main cause of fibrotic diseases [21]. These harmful effects are known to cause various diseases, including cancer, metabolic disorders, chronic inflammation, cardiovascular diseases, and neurodegenerative diseases (Figure 1) [22,23,24].

## 3. Dysregulation of Redox Homeostasis in Neurodegenerative Diseases

ROS, at lower levels, generally act as a small molecule messenger for cell signaling in the brain, whereas, at higher levels, they induce protein misfolding and neuronal cell death [18,25,26]. The brain requires large amounts of oxygen for mitochondrial respiration, which inevitably generates ROS. Conversely, the brain is very susceptible to excessive formation of ROS, as it is rich in polyunsaturated fatty acids, which are the preferred substrates of ROS, and it shows a low expression of antioxidants such as GSH [27,28]. Therefore, it was recognized that oxidative stress contributes to the pathogenesis of neurodegenerative diseases [24,29].

Although it is unclear whether it is the cause or result, a decrease in antioxidant levels and an increase in ROS levels are commonly observed in the nervous systems of patients with neurodegenerative diseases as well as animal models of such diseases, including Parkinson’s disease, Alzheimer’s disease, and amyotrophic lateral sclerosis [30,31,32,33,34]. Furthermore, aging, the major risk factor for most neurodegenerative diseases, induces loss of mitochondrial function and an imbalance in GSH homeostasis [35,36]. As a result, aging-induced pro-oxidizing states in the mitochondria and cells cause synaptic dysfunction, such as impairment of synaptic activity and neurotransmission [37]. In particular, increased ROS levels results in the thermodynamically unstable redox states of proteins, leading to their misfolding [38]. Various studies reported that oxidative stress triggers the formation of malfunctional proteins in the central nervous system, leading to abnormal release of neurotransmitters, and the formation of protein aggregates, which is a hallmark of several neurodegenerative diseases [26,39].

### 3.1. Alzheimer’s Disease

Alzheimer’s disease (AD), the most common neurodegenerative disease, is a progressive brain disorder that accounts for approximately 70% of dementia cases. AD is mainly characterized by the formation of extracellular amyloid-beta (Aβ) plaques, neurofibrillary tau tangles, and neuronal loss. Disruption of redox homeostasis was also observed in patients with AD [23,27]. Brain tissues or cells derived from patients with AD showed increased levels of ROS as well as oxidized proteins, and defects in antioxidant defense mechanisms [40,41,42,43]. Furthermore, the APP/PS1 double transgenic mice, which is a commonly used animal model of AD, showed a significant increase in ROS levels, which resulted in reduced neurogenesis in the brain [44]. In addition, increased ROS levels by H_2_O_2_ or NO caused AD pathology, such as the occurrence of brain lesions and memory deficits [45,46].

The formation of Aβ plaques or tau neurofibrillary tangles in the brain appears to be directly related to the impairment of redox homeostasis. Aβ, a small peptide derived from the cleavage of amyloid precursor protein (APP) by BACE1 and γ-secretase, forms oligomeric fibrils in the brains of patients with AD. As mentioned above, ROS facilitate the formation of neurofibrillary tangles by promoting the misfolding and cross-linking of proteins [23,47,48]. Some studies reported the potential role of Aβ monomers as antioxidants, depending on the metal ion concentration in vitro [49,50]. In contrast, other studies showed that oligomeric Aβ, the dominant form in patients with AD, promotes ROS production in cooperation with metal ions in vitro and in vivo [51,52,53,54]. In addition, inhibition of the γ-secretase activity was reported to reduce the levels of ROS and oxidize proteins, and to enhance resistance to oxidative stress [55]. Aβ activates NADPH oxidase and interferes with Ca^2+^ homeostasis and mitochondrial membrane potential, leading to impaired ROS homeostasis [53,56,57,58]. Tau neurofibrillary tangles, which contain oxidized molecules, also produce ROS by interacting with Cu^2+^, which in turn increases the formation of tau aggregates [59]. Furthermore, tau aggregation activates NADPH oxidase, which accelerates ROS-mediated neuronal death [60].

### 3.2. Parkinson’s Disease

Parkinson’s disease (PD) is a chronic neurodegenerative disorder that mainly affects the motor system; its primary symptoms are rigidity, tremor, bradykinesia, and postural instability. PD is characterized by the loss of dopaminergic neurons in the substantia nigra and accumulation of abnormal α-synuclein aggregates called Lewy bodies. The neuropathological characterization of PD appears to be directly related to the production of ROS, owing to mitochondrial dysfunction, neuroinflammation, and genetic mutations. Brain tissues derived from patients with PD showed defects in mitochondrial complex I and an increase in the levels of oxidized proteins [61]. Conversely, a systemic partial defect in the mitochondrial complex I is sufficient to reproduce the neuropathological features observed in patients with PD. Mutations in DJ-1 cause autosomal recessive forms of PD. Oxidized DJ-1 was observed in the brains of patients with idiopathic PD [62]. DJ-1 knock-out (KO) mice exhibited a significant increase in α-synuclein levels in the substantia nigra, increased levels of oxidized dopamine, and diminished the activity of glucocerebrosidase [63]. A reduction in GSH levels, which causes redox imbalance, was observed in the substantia nigra of patients with PD [64].

Oxidative stress is thought to be directly linked to PD pathogenesis. First, oxidative stress modifies and disrupts the activity of glucocerebrosidase, encoded by GBA1, whose mutations are commonly known genetic risk factors involved in the development of PD [65]. Second, α-synuclein accumulation appears to be directly affected by oxidative stress. α-Synuclein binds to ubiquitin and forms Lewy bodies. Oxidative stress leads to post-translational modifications of α-synuclein, such as HNE-α-synuclein and n-α-synuclein, which are more toxic than unmodified α-synuclein and are prone to form oligomers [66]. In particular, dopaminergic neurons in PD are susceptible to oxidative stress, as the dopamine metabolism generates ROS [67]. Levels of oxidized cholesterol metabolites derived from ROS were found to be increased in the cortex of patients with PD, resulting in accelerated generation of α-synuclein aggregates [68]. Brain tissues derived from patients with PD also showed oxidized nucleic acids, lipids, and proteins generated due to oxidative damage, indicating that oxidative damage of nucleic acids is probably a major risk factor for PD [69]. It is plausible that oxidative stress can trigger mutations resulting in cellular dysfunction, thereby increasing the chance of spontaneous mutations induced by this process [70]. The cytokine tumor necrosis factor (TNF), a critical regulator of the immune system, can contribute to neurodegeneration by promoting the generation and release of ROS or by exacerbating ROS generation by activating NADPH oxidase [71].

### 3.3. Amyotrophic Lateral Sclerosis

Amyotrophic lateral sclerosis (ALS) is a progressive neurological disease characterized by the death of motor neurons and leads to the impairment of voluntary muscle movement. Sporadic or familial ALS cases are caused by diverse gene mutations, including those in TAR DNA-binding protein 43 (TDP-43) and SOD1, the antioxidant enzyme [72]. SOD1 catalyzes the conversion of O_2_^●−^ to H_2_O_2_ with Cu^2+^. Therefore, the autosomal dominant mutations in SOD1, which account for up to 20% of familial ALS, increase the oxidative stress prior to the onset of ALS symptoms [73,74,75]. In addition, increased SOD1 activity boosted the production of ROS in the mitochondrial intermembrane space, resulting in morphological and functional impairment of the mitochondria [76,77]. Cells bearing the SOD1-G93A mutation are more vulnerable to ROS exposure, leading to the death of motor neurons [76,78]. Conversely, inhibition of SOD1 activity or treatment with antioxidants ameliorates the toxicity of motor neurons [76,79]. This increase in oxidative stress in cells caused SOD1 and TDP-43 protein aggregates, which are hallmarks of ALS [80,81]. As a result, the brain and motor neurons of patients with ALS as well as those of animal models of ALS exhibited increased levels of oxidized proteins, lipid peroxidation, and ROS, and decreased levels of antioxidants [82,83,84,85,86,87]. Furthermore, antioxidant treatment showed protective effects against neuronal survival and motor neuron impairment and decreased the ALS score [88,89].

## 4. Upregulation of Autophagy: An Effective Strategy to Maintain Redox Homeostasis in the Brain

### 4.1. General Description of Autophagy

Autophagy is a major intracellular degradation process that is important for the survival and homeostasis of cells [90,91]. Stress-induced autophagy mediates the clearance of protein aggregates, damaged intracellular organelles, and ROS. Autophagy is induced by a variety of stress stimuli such as starvation, ER stress, hypoxia, mitochondrial dysfunction, and oxidative stress. Notably, ROS were reported to be crucial early inducers of autophagy, as they are indispensable for the recruitment of LC3 into autophagosomes [92]. Autophagy is initiated by the formation and expansion of the phagophore to form the autophagosome, a double-membrane vesicle that sequesters cargoes to be degraded [93]. Autophagosomes then fuse with lysosomes to form autolysosomes, and the materials contained within them are degraded by the lysosomal enzymes. The degradation of cargoes by lysosomes generates nutrients that enable cells to survive [94]. After degradation, new lysosomes are formed from existing autolysosomes through autophagic lysosome reformation (ALR), a recently described lysosome biogenesis mechanism [95].

Increasing evidence suggests that autophagic impairments are closely linked to the pathogenesis of neurodegenerative disorders [96]. Mutations in autophagic receptors, such as p62 and optineurin (OPTN), are associated with a variety of neurodegenerative diseases, including PD, AD, and ALS [97,98]. The autophagic receptors are required for the interaction and autophagosomal recruitment of specific cargoes for degradation. Accumulation of protein aggregates, mostly due to impaired autophagic degradation, is a common feature of neurodegenerative diseases. Therefore, it is reasonable that the malfunction of the autophagic receptors is related to the etiology of neurodegenerative diseases. Conversely, diverse genes associated with neurodegenerative diseases were shown to function in autophagy [96,99]. For example, PTEN-induced kinase 1 (PINK1) and parkin, which are often mutated in patients with early onset autosomal recessive PD, are well-known to be critical for mitophagy, a selective autophagy that degrades damaged mitochondria [100]. Presenilin-1, which is associated with familial AD, regulates the maturation of lysosomal v-ATPase and affects the autophagic degradation of cargoes [101].

### 4.2. Regulatory Mechanism of ROS-Mediated Autophagy

**Figure 2:**
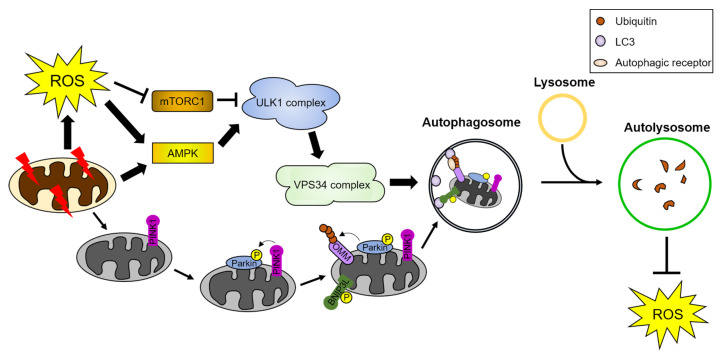
ROS induce autophagy and mitophagy. Damaged mitochondria and cellular stresses generate ROS. ROS activate AMPK and inhibit mTORC1, leading to sequential activation of the autophagy initiation complex, the ULK1 and VPS34 complex. The activated autophagy initiation complex induces autophagosome formation. Autophagosomes engulf cargoes, such as damaged organelles and proteins, and fuse with lysosomes. Autolysosomes degrade the autophagic cargoes. Damaged mitochondria are targeted for degradation by a form of selective autophagy, called mitophagy. PINK1 is recruited to damaged mitochondria and phosphorylates parkin, an E3 ubiquitin ligase, and mitophagy receptors such as BNIP3L/NIX. Parkin-mediated poly-ubiquitination of OMM proteins leads to the recognition of damaged mitochondria by autophagic receptors. These autophagic receptors interact with the ATG8 family proteins and transport the damaged mitochondria to autophagosomes for their degradation. Detailed pathway is described in Section 4. AMPK: AMP-activated protein kinase, BNIP3L: Bcl2 interacting protein 3 like, mTORC1: mammalian target of rapamycin complex 1, OMM: outer mitochondria membrane proteins, and PINK1: PTEN-induced kinase 1.

Several studies demonstrated that oxidation of cellular molecules by ROS, such as peroxidation of unsaturated lipids and DNA or the production of mitochondrial ROS, is sufficient for activating autophagy, which can mitigate the cytotoxic stress caused by ROS (Figure 2) [102,103,104]. Conversely, inhibition of autophagy by lysosomal inhibitors causes an increase in ROS-induced cytotoxicity [105,106]. ROS can directly induce autophagy by activating protein kinases, including AMP-activated protein kinase (AMPK) and c-Jun N-terminal kinase (JNK), which are essential for the activation of autophagy [107,108,109]. For example, ROS directly oxidize Cys299/Cys304 of AMPK and activate its kinase activity without reducing the cellular ATP levels [110]. Mitochondrial ROS production also activates AMPK and triggers an antioxidant response [111,112]. In addition, ROS activate JNK by stimulating the ER stress response [102]. ROS can also influence autophagy via the oxidation of core autophagy proteins, such as ATG4 and TFEB [113,114].

#### 4.2.1. ROS-Mediated Regulation of Mitophagy

Mitochondrial dysfunction is strongly associated with various diseases, including neurodegenerative disorders. Therefore, it is important to minimize mitochondrial dysfunction to counteract the development of numerous human diseases [115]. Mutations in genes encoding mitochondrial quality control proteins were found in patients with PD, indicating their failure to protect against stress-induced mitochondrial dysfunction [116]. Dysfunctional mitochondria can be degraded by mitophagy. Engulfment of mitochondria by autophagosomes can occur under various conditions, including starvation and oxidative stress, and when the mitochondrial function is impaired [117,118]. ROS produced by mitochondria act as signaling molecules for stress-induced autophagy, and ROS produced by damaged mitochondria induce mitophagy for their elimination [118].

In response to ROS, parkin, an E3 ubiquitin ligase, is selectively recruited to damaged mitochondria, promoting mitochondrial degradation [119]. PINK1, a mitochondrial serine/threonine-protein kinase, is required for the recruitment of parkin to the damaged mitochondrial membrane and activates parkin ligase activity [120]. Parkin mediates the formation of poly-ubiquitin chains in clustered mitochondria. Subsequently, p62, the autophagic receptor that recognizes poly-ubiquitin chains, is recruited to the ubiquitinated mitochondria for their clearance [121]. Moreover, VDAC1, a voltage-gated channel protein in the mitochondria, was identified as a mitophagy target through parkin-mediated-ubiquitylation. By interacting with LC3, mitophagy receptors, such as p62, mediate the targeting of damaged mitochondria into autophagosomes, for degradation [122]. Furthermore, BNIP3L/NIX, a BH3-only member of the BCL2 family, serves in mitochondrial clearance as a receptor that targets organelles for mitophagy [123].

#### 4.2.2. Transcriptional and Post-Transcriptional Regulation of Autophagy by ROS

TFEB is a master regulator of lysosomal biogenesis and autophagy [124]. Inactivated TFEB is retained in the cytoplasm, whereas activated TFEB translocates to the nucleus and promotes the transcription of genes essential for the autophagy–lysosome system. ROS directly oxidize the Cys212 residue of TFEB [113]. This oxidation inhibits the interaction of TFEB with RRAG GTPases, which promotes the translocation of TFEB to the nucleus, ultimately leading to the upregulation of lysosomal and autophagic pathways. ROS also induce transient receptor potential mucolipin 1 (TRPML1) channel-mediated lysosomal Ca^2+^ release [125]. Increased Ca^2+^ levels in the cytoplasm stimulate TFEB activity, which in turn promotes mitochondrial protein transcription, thereby reducing mitochondrial fragmentation, and recovering mitochondrial function [126,127]. Furthermore, overexpression of TFEB stabilizes Nrf2 by inhibiting DDB1 and CUL4-associated factor 11 (DCAF11), an E3 ligase of Nrf2, which enhances the transcription of Nrf2-response antioxidant genes, such as heme oxygenase-1 (HO-1), SOD2, and glutathione-s-transferase µ1 (GSTM1) [128].

Beclin-1, a component of the autophagy initiation complex (the VPS34 complex) that is essential for autophagy initiation, is regulated by various post-translational modifications [93,129]. ROS increase the protein levels and activity of Beclin-1 through the pro-apoptotic protein Bcl-2 [130,131]. Under normal conditions, Bcl-2 inhibits Beclin-1 activity via a direct binding to the BH domain within Beclin-1 [132,133]. Conversely, nutrient deprivation stimulates the JNK1-mediated phosphorylation of Bcl-2, leading to interference with its binding to Beclin-1 [134]. ROS decrease Bcl-2 protein levels through JNK-mediated Bcl-2 phosphorylation [135,136]. As a result, ROS increase Beclin-1 protein levels and promote autophagy initiation while simultaneously alleviating oxidative stress caused by ROS [130,131].

**Figure 3:**
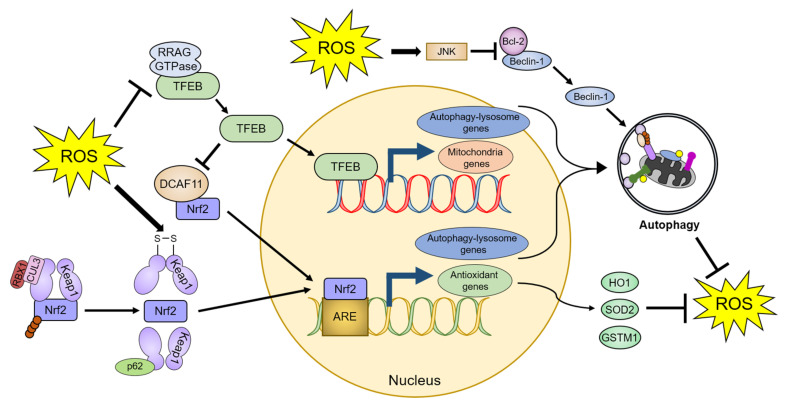
Autophagy-mediated regulation of ROS homeostasis. ROS promote autophagic activity by dissociating TFEB from RRAG GTPase and Beclin-1 from Bcl-2. ROS also activate Nrf2 through a conformational change in Keap1, leading to the dissociation of the Keap1-Nrf2 complex. Therefore, cells can ameliorate increased oxidative stress by the TFEB- and Nrf2-dependent upregulation of autophagic activity and antioxidant proteins. The detailed defense mechanisms are described in Section 4. TFEB: transcription factor EB, DCAF11: DDB1 and Cul4 associated factor 11, RBX1: Ring-box 1, CUL3: Cullin 3, ARE: antioxidant response element, JNK: c-Jun N-terminal kinase, HO1: Heme oxygenase 1, SOD2: Superoxide dismutase 2, and GSTM1: Glutathione-S-transferase µ1.

p62 is a representative autophagic receptor that recognizes ubiquitin-positive protein aggregates or mitochondria [137]. However, recent studies reported that p62 has a novel regulatory role in the Nrf2-Keap1 pathway, under oxidative stress conditions [138,139]. Nrf2 is a transcription factor that regulates several genes that encode antioxidants. Under normal conditions, the E3 ubiquitin ligase cullin 3 (CUL3) and ring-box 1 (RBX1) ubiquitinate Keap1, which interacts with Nrf2, leading to proteasomal degradation of Nrf2 [140]. Oxidative stress causes structural changes in Keap1 by generating an internal disulfide bond, resulting in the dissociation of Nrf2. The released Nrf2 binds to an antioxidant response element (ARE) sequence in target genes and suppresses the ROS-mediated toxicity [141,142]. p62 also interacts with Keap1 through the Kelch domain of Keap1, which is the Nrf2-binding site, and inhibits Keap1-mediated Nrf2 repression by producing Keap1 protein aggregates that are degraded by the proteasomal or autophagy–lysosomal system [138,143,144,145]. As the promoter of p62 contains an ARE sequence, activated Nrf2 can promote p62 transcription in response to oxidative stress [145]. The increased p62 levels can further activate Nrf2 [145,146]. Furthermore, since many promoters of autophagy core proteins, such as ATG5, ATG7, ATG9, ATG16L1, GABARAPL1, LAMP2A, and ULK1, contain ARE sequences, the brain tissues of Nrf2-deficient mice exhibit reduced protein levels of various autophagy regulators in response to ROS [147,148]. As a result, p62 and Nrf2 form a positive feedback loop to attenuate oxidative stress through activation of autophagy (Figure 3).

## 5. Autophagy as a Promising Therapeutic Strategy for Neurodegenerative Diseases

As described above, an imbalance in redox homeostasis contributes to the pathology of various neurodegenerative diseases, including AD, PD, and ALS, which exhibit increased ROS levels, as well as decreased levels of antioxidant proteins [37,42,76,87,149]. Various studies showed that adequate relief from oxidative stress can ameliorate the symptoms of neurodegenerative diseases, such as motor neuron death and cognitive dysfunction [45,53,76,149,150,151]. Therefore, induction of autophagy to eliminate ROS might be an appropriate therapeutic strategy for neurodegenerative diseases.

Treatment with small molecules can induce autophagy in an mTOR-dependent or -independent manner. Rapamycin, a representative autophagy inducer, inhibits the kinase activity of mTORC1. Rapamycin treatment reportedly reduced ROS production and improved abnormalities in neurodegenerative disease models [151,152,153]. For example, rapamycin treatment showed a protective effect by reducing intracellular ROS, as well as increasing antioxidant protein levels in vitro and in vivo [152,153]. Furthermore, rapamycin treatment recovered impaired synaptic function, neurotransmission, and cognitive ability, by reversing impaired redox homeostasis in animal models of AD [151].

Excessive use of mTOR-dependent autophagy inducers can cause various side effects, as mTOR signaling is important for global translation. Trehalose induces autophagy by activating AMPK or TFEB in an mTOR-independent manner [154]. It was suggested that trehalose might exhibit a protective effect against oxidative stress by scavenging ROS via the upregulation of SOD activity in plants [155]. In mammalian cells, trehalose causes the dissociation of Nrf2 from Keap1 through upregulation of the p62-mediated Nrf2 positive feedback loop [156]. Trehalose also promotes autophagy, especially mitophagy, resulting in the amelioration of oxidative stress [157].

Since ROS directly affect the function of autophagy genes associated with various neurodegenerative diseases, upregulation of autophagy genes is considered to be a preventive and therapeutic strategy for controlling these diseases. Therefore, several studies examined whether genetic activation of autophagy genes could relieve oxidative stress and the symptoms of neurodegenerative diseases in animal models.

Overexpression of TFEB enhances the degradation of substrates, lipid droplets, and damaged mitochondria, and alleviates the phenotypes associated with neurodegenerative diseases, such as PD and AD, by promoting autophagy and lysosomal biogenesis [124]. Moreover, adeno-associated virus (AAV)-mediated overexpression of TFEB was reported to show neuroprotective effects in rat nigral dopaminergic neurons [158]. Notably, the injection of viral vectors induced local protection of neuronal cell bodies and improved dopamine neurotransmission.

In response to ROS stimulation, CHK2 binds to Beclin-1 and phosphorylates it at Ser90/Ser93, thereby promoting autophagy via Beclin-1 release from Bcl-2 sequestration [159]. Beclin-1-deficient mice showed disrupted neuronal autophagy, abnormal APP processing, and increased neurodegeneration [160]. Lentivirus-mediated Beclin-1 transfer into the brain of α-synuclein transgenic mice relieved the synaptic and dendritic pathology with reduced α-synuclein accumulation [161]. Therefore, upregulation of Beclin-1 levels might have therapeutic potential in AD and PD.

Parkin functions as a critical regulator of mitophagy. Mutations in the parkin gene are linked to an autosomal recessive form, known as autosomal recessive juvenile parkinsonism [162]. Parkin overexpression by lentivirus ameliorated the dopaminergic neuronal cell death induced by α-synuclein overexpression in the rat substantia nigra [163]. In addition, parkin overexpression, using recombinant AAV (rAAV) in striatum of macaque monkeys, reduced the accumulation of co-expressed α-synuclein [164]. Therefore, parkin gene therapy is thought to be effective against α-synucleinopathy, suggesting its potential suitability for patients with PD [165].

DJ-1 is known to orchestrate a defense mechanism against oxidative stress, as its deletion leads to increased mitochondrial oxidative stress [166]. Dopamine adducts can modify α-synuclein and promote its aggregation; a recent study showed that loss of DJ-1 function is associated with Lewy body pathology [167]. As described earlier, DJ-1 KO mice showed increased levels of α-synuclein aggregation in the substantia nigra, accompanied by increased levels of oxidized dopamine [63]. Conversely, overexpression of DJ-1 in vitro effectively reduced the α-synuclein levels [168,169].

## 6. Conclusions

In this review, we discussed the intersection between redox homeostasis and autophagy, focusing on the pathogenesis of neurodegenerative diseases. Increasing evidence further clarifies that the disruption of redox homeostasis due to various reasons is a major cause of neurodegenerative diseases. When redox homeostasis is disrupted, cells upregulate antioxidant responses to restore it. Various studies revealed the potential role of autophagy in exerting a protective effect against oxidative stress, along with other antioxidant responses. In this context, it is necessary to elucidate the regulatory roles of autophagy in redox homeostasis in greater details. It is expected that optimal upregulation of autophagy could be a desirable therapeutic strategy to ameliorate neurodegenerative diseases.
